# Comparison of Predictive* In Silico* Tools on Missense Variants in* GJB2*,* GJB6*, and* GJB3* Genes Associated with Autosomal Recessive Deafness 1A (DFNB1A)

**DOI:** 10.1155/2019/5198931

**Published:** 2019-03-20

**Authors:** Vera G. Pshennikova, Nikolay A. Barashkov, Georgii P. Romanov, Fedor M. Teryutin, Aisen V. Solov'ev, Nyurgun N. Gotovtsev, Alena A. Nikanorova, Sergey S. Nakhodkin, Nikolay N. Sazonov, Igor V. Morozov, Alexander A. Bondar, Lilya U. Dzhemileva, Elza K. Khusnutdinova, Olga L. Posukh, Sardana A. Fedorova

**Affiliations:** ^1^Department of Molecular Genetics, Federal State Budgetary Scientific Institution “Yakut Science Centre of Complex Medical Problems”, Yakutsk, Russia; ^2^Laboratory of Molecular Biology, Institute of Natural Sciences, M.K. Ammosov North-Eastern Federal University, Yakutsk, Russia; ^3^Institute of Chemical Biology and Fundamental Medicine, Siberian Branch of the Russian Academy of Sciences, Novosibirsk, Russia; ^4^Novosibirsk State University, Novosibirsk, Russia; ^5^Laboratory of Human Molecular Genetics, Institute of Biochemistry and Genetics, Ufa Scientific Centre, Russian Academy of Sciences, Ufa, Russia; ^6^Department of Immunology and Human Reproductive Health, Bashkir State Medical University, Ufa, Russia; ^7^Department of Genetics and Fundamental Medicine, Bashkir State University, Ufa, Russia; ^8^Federal Research Center Institute of Cytology and Genetics, Siberian Branch of the Russian Academy of Sciences, Novosibirsk, Russia

## Abstract

*In silico* predictive software allows assessing the effect of amino acid substitutions on the structure or function of a protein without conducting functional studies. The accuracy of* in silico* pathogenicity prediction tools has not been previously assessed for variants associated with autosomal recessive deafness 1A (DFNB1A). Here, we identify* in silico* tools with the most accurate clinical significance predictions for missense variants of the* GJB2 *(Cx26),* GJB6 *(Cx30), and* GJB3* (Cx31) connexin genes associated with DFNB1A. To evaluate accuracy of selected* in silico* tools (SIFT, FATHMM, MutationAssessor, PolyPhen-2, CONDEL, MutationTaster, MutPred, Align GVGD, and PROVEAN), we tested nine missense variants with previously confirmed clinical significance in a large cohort of deaf patients and control groups from the Sakha Republic (Eastern Siberia, Russia): Сх26: p.Val27Ile, p.Met34Thr, p.Val37Ile, p.Leu90Pro, p.Glu114Gly, p.Thr123Asn, and p.Val153Ile; Cx30: p.Glu101Lys; Cx31: p.Ala194Thr. We compared the performance of the* in silico* tools (accuracy, sensitivity, and specificity) by using the missense variants in* GJB2* (Cx26),* GJB6* (Cx30), and* GJB3* (Cx31) genes associated with DFNB1A. The correlation coefficient (*r*) and coefficient of the area under the Receiver Operating Characteristic (ROC) curve as alternative quality indicators of the tested programs were used. The resulting ROC curves demonstrated that the largest coefficient of the area under the curve was provided by three programs: SIFT (AUC = 0.833,* p *= 0.046), PROVEAN (AUC = 0.833,* p *= 0.046), and MutationAssessor (AUC = 0.833,* p *= 0.002). The most accurate predictions were given by two tested programs: SIFT and PROVEAN (Ac = 89%, Se = 67%, Sp = 100%,* r *= 0.75, AUC = 0.833). The results of this study may be applicable for analysis of novel missense variants of the* GJB2 *(Cx26),* GJB6 *(Cx30), and* GJB3 *(Cx31) connexin genes.

## 1. Introduction

The most common form of hereditary nonsyndromic hearing loss is autosomal recessive deafness 1A (DFNB1A, MIM#220290) caused by pathogenic variants in the* GJB2*,* GJB6*, and* GJB3* genes encoding connexin 26 (Cx26), connexin 30 (Cx30), and connexin 31 (Cx31) proteins, respectively. The estimated prevalence of DFNB1A among general human population is 14:100 000, and the main cause of DFNB1A is biallelic recessive pathogenic variants in the* GJB2* gene (MIM#121011) (http://www.ncbi.nlm.nih.gov/books/NBK1272/, 2018). Currently, about 400 different pathogenic variations of* GJB2* sequence (more than 70% are missense or nonsense amino acid substitutions) are presented in the Human Gene Mutation Database (HGMD, http://www.hgmd.cf.ac.uk/ac/all.php), and this list is regularly updated by novel yet unclassified variants. The majority of nonsense variants are pathogenic since they lead to a premature termination of translation and protein synthesis, while missense variants depending on their location in the amino acid sequence can be neutral, damaging, or partially damaging to the structure and function of protein. As a consequence, pathogenicity of many missense variants is difficult to assess.

Basic information on pathogenic mutations is provided by curated databases such as Online Mendelian Inheritance in Man (OMIM) [1] and the Human Gene Mutation Database (HGMD) [2] collecting data on variants of all genes, mainly from the literature. Disease and gene-specific databases often contain variants that are incorrectly classified including incorrect claims published in peer-reviewed literature since different authors interpret the term “mutation pathogenicity” differently because of the increased complexity of analysis and interpretation of clinical genetic testing. Experimental study of the molecular effects of mutations is laborious, whereas useful and reliable information about the effects of amino acid substitutions can readily be obtained by theoretical methods [3]. A variety of* in silico* tools, both publicly and commercially available, can help in the interpretation of sequence variants without structural or functional studies. However, algorithms used by each tool may differ, but can include determination of the effect of the sequence variant at the nucleotide and amino acid as well as the potential impact of the variant on the protein. The impact of a missense substitution depends on criteria such as the evolutionary conservatism of an amino acid/nucleotide, location, and context within the protein sequence and the biochemical consequence of the amino acid substitution [4].

Different* in silico* tools each have their own strengths and weaknesses depending on the algorithm, and in many cases performance varies depending on the certain gene and protein [5, 6]. Performance of available prediction software is constantly being evaluated by comparing their ability to predict “known” disease-causing variants. As a result, the MutPred performed best for variants of genes associated with the RASopathy and limb-girdle muscular dystrophy (LGMD) [7]; the MAPP and the MAPP + PolyPhen-2.1 provided the best combined model for testing variants of* MLH1*,* MSH2*,* MSH6*, and* PMS2* genes associated with Lynch syndrome, a hereditary form of colon cancer [8]; the SIFT was well suited for the analysis of variants of the* UGT1A1* gene associated with Crigler-Najjar syndrome (congenital hereditary nonhemolytic unconjugated bilirubinemia) [9]; the Align GVGD* in silico* tool was shown as the best for testing variants of genes associated with cancer (*BRCA1*,* BRCA2*,* MLH1*, and* MLH2*) [10];* in silico *test of 236* BRCA1/2* missense variants suggested that SIFT and MutationTaster2 are suitable to predict benignity of variants in these genes [11]. There is also a big class of tools for predicting splice site variations which were tested by comparing the predictions against RNA* in vitro* results for natural splice sites of clinically relevant genes in hereditary breast/ovarian cancer (HBOC) [12]. The analysis revealed that HSF, HSF+SSF-like, or HSF+SSF-like+MES achieved a high performance for predicting the disruption of donor sites, and SSF-like for predicting disruption of acceptor sites [12]. In general, most missense variant prediction algorithms are 65-90% accurate when examining known disease variants.

However, so far the accuracy of* in silico* pathogenicity prediction tools was not assessed for variants of genes associated with autosomal recessive deafness 1A. To date, the only published study was focused on the pathogenicity analysis of 211 missense variants of the* GJB2* gene annotated in the Ensembl and the HGMD databases [13]. Four predictive* in silico* tools, SIFT, PANTHER, PolyPhen-2, and FATHMM, were used but the comparison of their performance was not performed.

The aim of this study is to compare the performance of the* in silico *pathogenicity prediction tools by testing the missense variants in* GJB2* (Cx26),* GJB6* (Cx30), and* GJB3* (Cx31) genes associated with the autosomal recessive deafness 1A.

## 2. Materials and Methods

### 2.1. Missense Variants Selection

**Figure 1:**
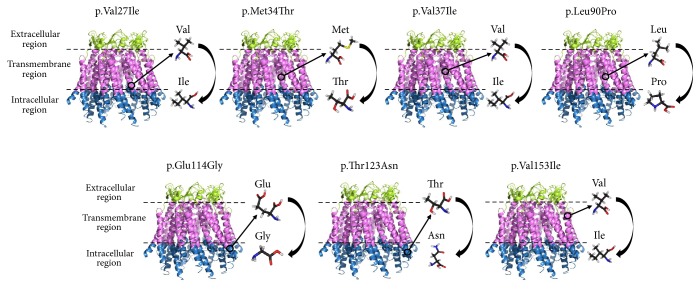
Localization of the tested nonsynonymous (missense) amino acid substitutions in the structure of connexin 26.* Note.* The information about the structure Сx26 was obtained from the database of three-dimensional structures of proteins and nucleic acids PDB ID:2ZW3 (https://www.ncbi.nlm.nih.gov/Structure/pdb/2ZW3) [22]. Localization of the studied amino acids in structure of Cx26 was obtained using the 3D-structure viewer applet with the protein structure loaded software PolyPhen-2 (http://genetics.bwh.harvard.edu/pph2/). Detailed structure models of human Cx30 and Cx31 proteins are currently not defined.

To assess accuracy of selected* in silico* tools, we tested nine missense variants of the* GJB2* (Cx26),* GJB6* (Cx30), and* GJB3* (Cx31) genes found earlier in a large cohort of deaf patients and control groups from the Sakha Republic (Eastern Siberia, Russia):* GJB2 *(Сх26): c.79G>A (p.Val27Ile), c.101T>C (p.Met34Thr), c.109G>A (p.Val37Ile), c.269T>C (p.Leu90Pro), c.341A>G (p.Glu114Gly), c.368C>A (p.Thr123Asn), and c.457G>A (p.Val153Ile);* GJB6* (Cx30): c.301G>A (p.Glu101Lys);* GJB3* (Cx31): с.580G>A (p.Ala194Thr) [14–16] (Figure 1). Of these, three variants of the* GJB2* gene, c.269T>C (p.Leu90Pro), c.101T>C (p.Met34Thr), and c.109G>A (p.Val37Ile), are pathogenic variants associated with hearing impairment (DFNB1A); the remaining six variants were interpreted as benign variants of no clinical significance [14, 15]. To assess the clinical relevance of the presented missense variants, we analyzed not only the results of the segregation analysis of genotype-phenotype correlation, but also the data from the databases of annotated variants: OMIM (the Online Mendelian Inheritance in Man, http://www.omim.org) [1]; HGMD (the Human Gene Mutation Database, http://www.hgmd.cf.ac.uk) [2]; the ClinVar (a public archive with interpretations of clinically relevant variants, http://www.ncbi.nlm.nih.gov/clinvar/) [17, 18]; ExAC (the Exome Aggregation Consortium, http://exac.broadinstitute.org) [19]; the 1000 Genomes Project (http://www.ncbi.nlm.nih.gov/variation/tools/1000genomes) [20]; dbSNP (the Single Nucleotide Polymorphism database, http://www.ncbi.nlm.nih.gov/snp/) [21].

### 2.2. In Silico Prediction Tools

In this study, 9 predictive computer programs were used to predict pathogenicity: SIFT (Sorting Intolerant From Tolerant) [3, 24–27], FATHMM (Functional Analysis Through Hidden Markov Models) [28–30], MutationAssessor [31, 32], PolyPhen-2 (Polymorphism Phenotyping V-2) [33], CONDEL (Consensus Deleteriousness) [34], MutationTaster [35, 36], MutPred (Mutation Prediction) [37], Align GVGD (Align Grantham Variation/Grantham Deviation) [38, 39], and PROVEAN (Protein Variation Effect Analyzer) [40, 41]. Each* in silico* tool uses different parameters for classification of variants which are detailed according to websites listed in Supplementary Materials (see Table S1). The FASTA format and Ensembl sequence identifiers (nucleotide, amino acid, and protein) were used for query in programs (see Table S2).

### 2.3. Analytical Parameters of In Silico Tools

Analytical parameters of studied tools were calculated according to Fletcher & Fletcher, 2005, and Glantz, 1997 [23, 42]:

*Sensitivity (Se)* is a proportion of the true-positive results (correct identification of pathogenic variants), according to equation

(1) $$\begin{array}{c} Se=\frac{Tp}{Tp}+FN\times \text{100\%} \end{array}$$

where* Tp *denotes true-positive cases and* FN *denotes false negative cases.

*Specificity (Sp)* is a proportion of the true negative results (correct identification of benign variants), according to equation

(2) $$\begin{array}{c} Sp=\frac{TN}{TN}+Fp\times \text{100\%} \end{array}$$

where* TN *denotes true negative cases and* Fp *denotes false-positive cases.

*Accuracy (Aс) *is the ratio of complete correct predictions to the total number of predictions, according to the following equation.

(3) $$\begin{array}{c} Ac=Tp+\frac{TN}{Tp}+TN+Fp+FN\times \text{100\%} \end{array}$$

*Positive predictive values (PPV) *are a proportion of positive results that were true-positive (the ratio of true-positive results to all positive results), according the following equation.

(4) $$\begin{array}{c} PPV=\frac{Tp}{\text{T}p}+Fp\times \text{100\%} \end{array}$$

*Negative predictive values (NPV)* are a proportion of negative results that were true negative (the ratio of true negative results to all negative results), according to the following equation.

(5) $$\begin{array}{c} NPV=\frac{TN}{TN}+FN\times \text{100\%} \end{array}$$

*Correlation coefficient (r)* is the determination of the relationship between the clinical values of missense variants and predictive evaluation of the program.

*ROC curve*: the way to express the relationship between sensitivity and specificity for a given test is to construct a curve, called a Receiver Operating Characteristic (ROC) curve [42]. ROC curves are frequently used in the bioinformatic analysis to evaluate classification and prediction models for supporting, diagnosis, and prognosis. To construct a ROC curve, along the Y-axis, the true-positive share (sensitivity) is plotted, along the X-axis, the false-positive share (1 − specificity). The values on the axes ran from probability of 0 to 100% [42]. The quantitative interpretation of ROC is given by AUC (area under ROC curve), the area bounded by the ROC curve and the axis of the share of false-positive cases. The bigger the area under the ROC curve, the better the model. A rough guide for classifying the accuracy of a diagnostic test is the traditional academic point system: 0.9-1.0: excellent (A); 0.8-0.9: good (B), 0.7-0.8: fair (C); 0.6-0.7: poor (D); 0.5-0.6: fail (F) (corresponds to random guessing) [43]. The ROC curves were constructed using the MedCalc statistical software for biomedical research (https://www.medcalc.org).

## 3. Results

The predictions for missense variants in the* GJB2* (Cх26),* GJB6* (Сх30), and* GJB3* (Cx31) genes by the* in silico *tools in comparison with their established clinical significance are presented in Table 1. Predictions for studied missense variants (3 pathogenic, 6 benign) were different in every analyzed* in silico *tool. Only the c.269T>C (p.Leu90Pro) variant of the* GJB2* gene was evaluated by all programs as a damaging variant.

The informative parameters of the compared programs are presented in Table 2. The accuracy of the clinical significance predictions for missense variants among the analyzed nine programs varies from 33% (FATHMM) to 89% (SIFT and PROVEAN). The SIFT and PROVEAN showed high sensitivity and specificity parameters: 67% and 100%, respectively. The programs MutationAssessor, FATHMM, MutationTaster, and CONDEL had 100% sensitivity, but showed a low specificity, between 33% and 67%, and CONDEL showed total absence of specificity. High rates of predictability of positive and negative results were provided by the SIFT and PROVEAN programs (PPV = 100% and NPV = 86% for both programs) while the FATHMM and Align GVGD programs were the most inaccurate, which resulted in a decrease in almost all of the analyzed parameters. However, FATHMM showed 100% sensitivity since all missense variants were classified by this program as equally damaging.

**Figure 2:**
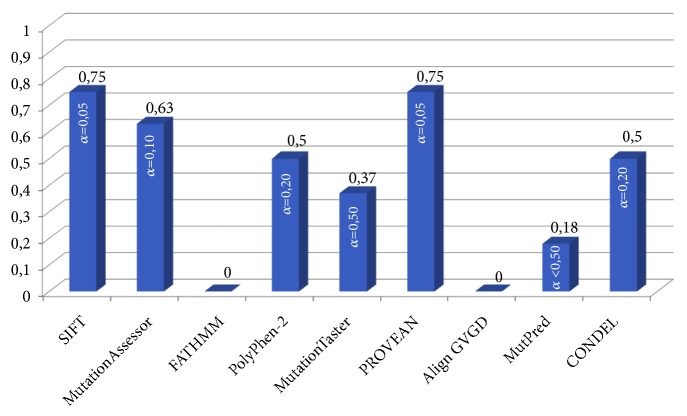
The correlation coefficient (*r*) histogram.* Note. r*: the relationship between the known clinical significance of missense variants and* in silico* evaluation given by 9 predictive tools; *α*: the level of significance of the correlation coefficient: the critical value for the significance level and the sample size n=9 is 0.933, so the correlation is significant at p<0.001 [23].

The overall correlation coefficients are presented in Figure 2. The SIFT and PROVEAN programs demonstrate the highest correlation of* in silico* predictions with observed clinical significance of missense substitutions (*r *= 0.75) which corresponds to their analytical parameters (Table 2). The average values of correlation were shown for MutationAssessor (*r *= 0.63), PolyPhen-2 (*r* = 0.5), and CONDEL (*r *= 0.5) which also correspond to their analytical parameters (Table 2). The MutationTaster demonstrated a weak correlation (*r*=0.37), MutPred showed very weak correlation (*r* = 0.18), and the FATHMM and Align GVGD programs showed no correlation between the observed values (*r *= 0).

**Figure 3:**
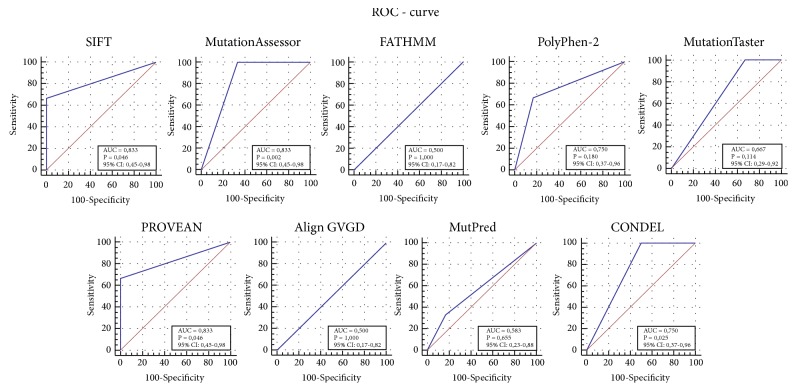
ROC curves expressing the relationship of the sensitivity and specificity of the tested programs. These graphs illustrate performance of studied* in silico* tools. The overall accuracy of the tests can be described as the area under the ROC curve (AUC); a higher AUC score indicates a better performance. The diagonal line shows the relationship between true-positive and false-positive values of absolutely uninformative* in silico* tools (FATHMM and Align GVGD). 95% CI indicates 95% confidence interval (Binomial Exact). The ROC curves were constructed using the MedCalc statistical software for biomedical researches (https://www.medcalc.org).

The result of ROC curve analysis is shown in Figure 3. The resulting ROC curves demonstrated that the largest coefficient of the area under the curve was shown by three programs: SIFT (AUC = 0.833,* p *= 0.046, 95% CI: 0.45-0.98), PROVEAN (AUC = 0.833,* p* = 0.046, 95% CI: 0.45-0.98), and MutationAssessor (AUC = 0.833,* p *= 0.002, 95% CI: 0.45-0.98). For PolyPhen-2 and CONDEL, the area of the curve was in the range of 0.7-0.8 (AUC = 0.750,* p* = 0.175, 95% CI: 0.37-0.96), and for MutationTaster it was in the range of 0.6-0.7 (AUC = 0.665,* p *= 0.114, 95% CI: 0.29-0.92). Two programs, FATHMM and Align GVGD, showed a complete lack of information in the predictions (AUC = 0.500,* p *= 1.000, 95% CI: 0.17-0.82).

## 4. Discussion

For the first time, we analyzed the informative parameters of nine predictive* in silico* tools, obtained by predictions of the clinical significance of missense variants of* GJB2* (Cx26),* GJB6* (Cx30), and* GJB3* (Cx31) connexin genes associated with hearing impairment. The capabilities of* in silico* prediction tools were demonstrated by testing nine missense variants with confirmed clinical significance of* GJB2* (Cх26),* GJB6* (Cx30), and* GJB3* (Cx31) genes detected earlier in the study of congenital hearing impairment in the Sakha Republic of Russia [14, 15]. The results of this study may be applicable for analysis of novel missense variants of the* GJB2* (Cx26),* GJB6* (Cx30), and* GJB3* (Cx31) genes.

We focused on nine programs chosen according to the following criteria: predicting the impact of missense variants on the function or structure of the protein, differing in computational methods and/or tools, popularity (the top programs included in the dbNSFP [44]), and free online access. Parameters such as accuracy, sensitivity, and specificity were chosen to assess their predictive abilities. Without these parameters, it is not possible to fully evaluate the accuracy of a test [42].

As a result, the SIFT and PROVEAN programs showed the highest sensitivity (Se = 67%) and specificity (Sp = 100%). Thus, the requirement for maximum total sensitivity and specificity in our study was 167% (Se + Sp), while the required balance between sensitivity and specificity was 33% (∆ Se - Sp). The accuracy (Ac) of the predictions of the SIFT and PROVEAN programs was 89%. This result can be considered as the best in this study; it can also be compared to accuracy of predictions published earlier in other studies: 80% - 90% [6, 7, 28, 36, 45]. A lower accuracy was shown by MutationAssessor (Ac = 78%), CONDEL (Ac = 67%), and MutationTaster (Ac = 56%) that were highly sensitive (Se = 100%), but not very specific (Sp = 33-67%). These results indicate a low accuracy of predictions for neutral variants. Align GVGD (Ac = 44%) and FATHMM (Ас = 33%) produced a large number of incorrect pathogenicity predictions and thus were unacceptable for testing variants of the studied genes.

In addition to the obtained characteristics of accuracy, sensitivity, and specificity, we also used correlation coefficients (*r*) and areas under the ROC curve (AUC) as alternative indicators of the quality of the tested programs. We compared the values of* r* and AUC with the quantitative values of the exact predictions of the* in silico* tools under study. For instance, the highest values of* r* = 0.75 were shown by the SIFT and PROVEAN programs that gave the highest number of correct predictions. As is known, the higher the predictive power of the model, the closer the ROC curve to the upper left corner, where the fraction of true-positive cases is 100% (ideal sensitivity) and the share of false-positive cases is zero [42]. The resulting ROC curves demonstrated that the curves of SIFT and PROVEAN were closest to the ideal chart, with the largest area under the curve: AUC = 0.83 (95% confidence interval is 0.45-0.98), which indicates a very good quality of predictions. The ROC curves of FATHMM and Align GVGD on the diagonal line indicated an absolute lack of informativeness (AUC = 0.500, which corresponds to random guessing); as a result, they had the most erroneous predictions. Our results confirmed that the best programs for bioinformatic analysis of missense variants of the* GJB2* (Cx26),* GJB6* (Cx30), and* GJB3* (Cx31) connexin genes are SIFT and PROVEAN.

The resulting performance of the PROVEAN and SIFT tools turned out to be fully comparable, as previously described [40, 41]. Note that both programs have the same algorithm of assessing variants by whether they occur in evolutionary conserved region or not, which uses the most popular service, BLASTP (Basic Local Alignment Search Tool) [3, 24, 27, 40, 41]. Thus, we can assume that both tools have the same predictability. However, it should be noted that SIFT predicts the effects of all possible substitutions at each position in the protein sequence calculated from a Dirichlet mixture. On the other hand, PROVEAN provides a generalized approach to predict the functional effects of protein sequence variations computed based on BLOSUM62 [40]. The obtained data indicate that, with a wide choice of predictive programs, it is important to consider their methods and tools used for analysis. Also, it should be considered that any computer analysis of biological data is an* in silico* experiment, which has only a more or less reliable prediction that must be verified by other comprehensive structural/functional studies.

## 5. Conclusion

In summary, the analysis of all obtained informative parameters (accuracy, sensitivity, and specificity) of the nine* in silico *tools along with the correlation coefficient and the area under the ROC curve showed that SIFT and PROVEAN were the tools with the best pathogenicity prediction power; MutationAssessor, PolyPhen-2, and CONDEL performed at an average level; MutationTaster and MutPred were below average; and Align GVGD and FATHMM were uninformative. The results of this study may be applicable for analysis of novel missense variants of the* GJB2* (Cx26),* GJB6* (Cx30), and* GJB3* (Cx31) genes.

## Figures and Tables

**Table 1:** Evaluation of missense variants by predictive *in silico* tools.

Gene	Missense variants	Clinical significance	SIFT	FATHMM	MutationAssessor	Polyphen-2	CONDEL	MutationTaster	MutPred	Align GVGD	PROVEAN
*GJB2* (Cx26)	c.79G>A p.Val27Ile rs2274084	Benign	**Tolerated** **score: 0.21**	Damaging score: -5.59	Medium FI score: 2.28 VC score: 2.16 VS score: 2.40	Probably damaging HumDiv score: 1.000 HumVar score: 0.998	Deleterious Calculated Condel score: 0.612278613903	**Polymorphism** **score: 29**	**hypotheses are absent** **general score: 0.321**	**Unclassified** **Class C25** **GV 0.00** **GD 29.61**	**Neutral** **score: -0.660**
	c.101T>C p.Met34Thr rs35887622	Pathogenic	**Damaging** **score: 0.01**	**Damaging** **score: -5.41**	**Medium** **FI score: 2.315** **VC score: 2.43** **VS score: 2.20**	Benign HumDiv score: 0.038 HumVar score: 0.083	**Deleterious** **Calculated Condel** **score:** **0.58786807751**	**Disease causing** **score: 81**	hypotheses are absent general score: 0.969	**Deleterious** **Class C65** **GV 0.00** **GD 81.04**	**Deleterious** **score: -3.801**
	c.109G>A p.Val37Ile rs72474224	Pathogenic	Tolerated score: 0.34	**Damaging** **score: -5.46**	**Medium** **FI score: 2.095** **VC score: 2.58** **VS score: 1.61 **	**Probably damaging** **HumDiv score: 1.000** **HumVar score: 0.996**	**Deleterious** **Calculated** **Condel score:** **0.61487213316**	**Disease causing** **score: 29**	hypotheses are absent general score: 0.902	Unclassified Class C25 GV 0.00 GD 29.61	Neutral score: -0.857
	c.269T>C p.Leu90Pro rs80338945	Pathogenic	**Damaging** **score: 0**	**Damaging** **score: -5.64**	**Medium** **FI score: 3.33** **VC score: 4.26** **VS score: 2.40**	**Probably** **damaging** **HumDiv score: 1.000** **HumVar score: 0.996**	**Deleterious** **Calculated Condel** **score:** **0.676708483818**	**Disease causing** **score: 98**	**Confident hypotheses:** **Gain of sheet** **(P = 0.039)** **general score:0.915**	**Deleterious** **C65** **GV 0.00** **GD 97.78**	**Deleterious** **score: -6.482**
	c.341A>G p.Glu114Gly rs2274083	Benign	**Tolerated** **score: 0.16**	Damaging score: -4.58	Medium FI score: 2.005 VC score: 2.40 VS score: 161	**Benign** **HumDiv score: 0.001** **HumVar score: 0.001**	Deleterious Calculated Condel score: 0.556433693212	**Polymorphism** **score: 98**	**hypotheses are absent** **general score: 0.232**	Deleterious Class C65 GV 0.00 GD 97.85	**Neutral** **score: -0.123**
	c.368C>A p.Thr123Asn rs111033188	Benign	**Tolerated** **score: 0.59**	Damaging score: -4.42	**Neutral** **FI score: -0.305** **VC score: -0.61** **VS score: - 0 **	**Benign** **HumDiv score: 0.000** **HumVar score: 0.000**	**Neutral** **Calculated Condel** **score:** **0.513276654484**	Disease causing score: 53	**hypotheses are absent** **general score: 0.201**	Deleterious Class C55 GV 0.00 GD 64.77	**Neutral** **score: 0.797**
	c.457G>A p.Val153Ile rs111033186	Benign	**Tolerated** **score: 1**	Damaging score: -3.69	**Neutral** **FI score: -0.305** **VC score: -0.43** **VS score: -0.18**	**Benign** **HumDiv score: 0.003** **HumVar score: 0.007**	**Neutral** **Calculated Condel** **score:** **0.491937780564**	Disease causing score: 29	**hypotheses are absent** **general score: 0.488**	**Unclassified** **Class C25** **GV 0.00** **GD 29.61**	**Neutral** **score: 0.138**

*GJB6* (Cx30)	c.301G>A p.Glu101Lys rs571454176	Benign	**Тolerated** **score:0.69**	Damaging score: -5.26	**Neutral** **FI score: -0.37** **VC score: -0.74** **VS score: 0 **	**Benign** **HumDiv score: 0.193** **HumVar score: 0.058**	**Neutral** **Calculated Condel** **score:** **0.505405538667**	Disease causing Score: 56	Actionable hypotheses: Gain of MoRF binding (P = 0.0064) Gain of ubiquitination at E101 (P = 0.0276) Gain of methylation at E101 (P = 0.0345) general score: 0.506	Deleterious Class C55 GV 0.00 GD 56.87	**Neutral** **score: -1.273**

*GJB3* (Cx31)	с.580G>A p.Ala194Thr rs121908852	Benign	**Тolerated** **score: 0.91**	Damaging score: -3.67	**Low** **FI score: 1.085** **VC score: -0.54** **VS score: 2.71**	**Benign** **HumDiv score: 0.163** **HumVar score: 0.110**	Deleterious Calculated Condel ** **score: 0.529626647419	Disease causing Score: 58	**hypotheses are absent** **general score: 0.399**	Deleterious Class C55 GV 0.00 GD 58.02	**Neutral** **score: 1.636**

*Note.* The correct results (both “true” positive and “true” negative results) are highlighted by bold font.

**Table 2:** Performance of *in silico* tools.

*in silico* Tools	Accuracy	Sensitivity	Specificity	PPV	NPV
SIFT	89%	67%	100%	100%	86%
MutationAssessor	78%	100%	67%	60%	100%
FATHMM	33%	100%	0%	33%	0%
Polyphen-2	78%	67%	83%	67%	50%
MutationTaster	56%	100%	33%	43%	33%
PROVEAN	89%	67%	100%	100%	86%
Align GVGD	44%	33%	33%	33%	67%
MutPred	67%	33%	83%	50%	71%
CONDEL	67%	100%	50%	50%	100%

*Note. Accuracy (Aс)* - the proportion of the correct test results (that is the sum of true positive and true negative results) among all the patients examined. In our case, this is the proportion of correct estimates of pathogenic and benign variants; *Sensitivity (Se)* - the ability of the diagnostic method to give the correct result which is defined as the proportion of true positive results among all performed tests. In our case, this is the proportion of true positive results, that is, the correct identification of pathogenic variants; *Specificity (Sp)* - the ability of the diagnostic method not to give false positive results in the absence of disease, which is defined as the proportion of true negative results among healthy individuals in studied group. In our case, this is a share of true negative results, that is, a correct identification of benign variants; *Positive predictive values (PPV)* - prediction of pathogenic variants; *Negative predictive values (NPV)* - prediction of benign variants.

## Data Availability

The data used to support the findings of this study are available from the corresponding author upon request.

## Supplementary Materials

Supplementary Materials Table S1: description of the* in silico* tools; Table S2: sequence identifiers.
