# Supplementary material for: Comparison of Predictive In Silico Tools on Missense Variants in GJB2, GJB6, and GJB3 Genes Associated with Autosomal Recessive Deafness 1A (DFNB1A)
Source: ScientificWorldJournal. 2019 Mar 20;2019:5198931. doi: 10.1155/2019/5198931 (PMC6446107; doi:10.1155/2019/5198931)
Supplement: Supplementary Materials — Table S1: description of the in silico tools; Table S2: sequence identifiers. [file 5198931.f1.pdf]

Supplementary Materials

Table S1: Description of the *in silico* tools.

Program (web-site)	Basis			Classification			References
	Algorithm	Method	Computing tools	Effect	Score	Prediction	
**SIFT** (http://sift.jcvi.org)	Evolutionary conservation	Compilation of a data set of functionally linked protein sequences using BLAST/PSI-BLAST	Matrix Dirichlet	Effect of amino acid substitution on structure/function of protein	0.00 - 1	<0.05 = "Damaging"<br>>0.05 = "Tolerated"	[3, 22-25]
**FATHMM** (http://fathmm.biocompute.org.uk/)			Hidden Markov Model (HMM)		> -1.5 <	<-1.5 = "Damaging"<br>>-1.5 = "Tolerated"	[26-28]
**MutationAssessor** (http://mutationassessor.org/)		Provides data from other databases such as COSMIC, UniProt and Pfam as well as its own "functional point of influence" on mutation	Cross-Entropy Method		-5.76 - 5.76	≤0.8 = "neutral"<br>0.8≤1.9 = "low"<br>1.9≤3.5 = "medium"<br>>3.5 = "high"	[29, 30]
**PolyPhen2** (http://genetics.bwh.harvard.edu/pph2/)	Protein structure/function and evolutionary conservation	Statistical method of weighting and profiling sequences from subsets of identical sequences in several alignments using PSIC	Naive Bayesian classifier		Two models:<br>HumDiv: 0.00 - 1<br>HumVar: 0.00 - 1	0.0 - 0.15 = "benign"<br>0.15 - 1.0 = "possibly damaging"<br>0.85 - 1.0 = "probably damaging"	[31]
**CONDEL** (http://bg.upf.edu/fannsdb/)		Combines SIFT, PolyPhen-2, MutationAssessor and FATHMM			0.00 - 1	0.0 = "Neutral"<br>1.0 = "Deleterious"	[32]
**MutationTaster** (http://www.mutationtaster.org/)	Protein structure/function and evolutionary conservation	Integration of information from various biomedical databases (Ensembl, UniProt, ClinVar, ExAC, 1000 Genomes Project, phyloP, phastCons)	Naive Bayesian classifier	Cause of disease	0.0 - 215 (does not affect forecast)	"disease causing"<br>"disease causing automatic"<br>"polymorphism"<br>"polymorphism automatic"	[33, 34]
**MutPred** (http://mutpred.mutdb.org/)	Protein structure/function and evolutionary conservation	Based on established SIFT method	Support Vector Machines (SVM)	Effect of amino acid substitution on structure/function of protein	g = 0.00 - 1 (g - total score) (p - rating 5 properties)	*g*>0.5, *p*<0.05 = "Actionable hypotheses"<br>*g*>0.75, *p*<0.05 = "Confident hypotheses"<br>*g*>0.75, *p*<0.01 = "Very confident hypotheses"	[35]
**Align GVGD** (http://agvgd.hci.utah.edu/agvgd_input.php)	Protein structure/function and evolutionary conservation	Measurement of biochemical distances between amino acids (norm/substitution), according to MSA	Matrix of Grantham GV (Grantham Variation) GD (Grantham Deviation)	Pathogenetic effect on protein structure/function of protein	GVGD = Class: C0, C15, C25, C35, C45, C55, C65	C65 - most likely<br>C0 - less likely	[36, 37]
**PROVEAN** (http://provean.jcvi.org/index.php)	Evolutionary Conservation/Alignment and measurement of similarity between variant sequence and protein sequence homolog	Compilation of a data set of functionally linked protein sequences using BLASTP, with further processing of large databases CD-HIT (ver.4.5.5)	Blocks Substitution Matrix (BLOSUM62)	Functional effect on protein	- 40 - 12.5 (threshold: -2.5)	≥- 2.5 = "deleterious"<br>≤- 2.5 = "neutral"	[38, 39]

Table S2: Sequence identifiers.

Database	Gene	Protein	Gene ID	Protein ID	Transcript ID
**NCBI Reference Sequence** https://www.ncbi.nlm.nih.gov/refseq/	*GJB2*	Cx26	NM_004004.5	NP_003995.2	NC_000013.11
	GJB6	Cx30	NM_001110219.2	NP_001103689	NC_000013.11
	GJB3	Cx31	NM_001005752.1	NP_001005752	NC_000001.11
**Ensembl ID** http://www.ensembl.org/Homo_sapiens/Gene/	*GJB2*	Cx26	ENSG00000165474	ENSP00000372299	ENST00000382848
	GJB6	Cx30	ENSG00000121742	ENSP00000241124.6	ENST00000241124.10
	GJB3	Cx31	ENSG00000188910	ENSP00000362460	ENST00000373366.2
